# Long-Term Impact of War on Healthcare Costs: An Eight-Country Study

**DOI:** 10.1371/journal.pone.0029603

**Published:** 2012-01-04

**Authors:** Ramon Sabes-Figuera, Paul McCrone, Marija Bogic, Dean Ajdukovic, Tanja Franciskovic, Niccolò Colombini, Abdulah Kucukalic, Dusica Lecic-Tosevski, Nexhmedin Morina, Mihajlo Popovski, Matthias Schützwohl, Stefan Priebe

**Affiliations:** 1 Centre for the Economics of Mental Health, Institute of Psychiatry, King's College London, London, United Kingdom; 2 Unit for Social & Community Psychiatry, Barts' and the London School of Medicine and Dentistry, Queen Mary University of London, London, United Kingdom; 3 Faculty of Humanities and Social Sciences, University of Zagreb, Zagreb, Croatia; 4 School of Medicine, University of Rijeka, Rijeka, Croatia; 5 Department of Mental Health, Modena, Italy; 6 School of Medicine, University of Sarajevo, Sarajevo, Bosnia and Herzegovina; 7 Belgrade University School of Medicine, Belgrade, Serbia; 8 Department of Clinical Psychology, University of Amsterdam, Amsterdam, Netherlands; 9 Institute of Psychology, University of Skopje, Skopje, FYR Macedonia; 10 Department of Psychiatry and Psychotherapy, Technische Universität Dresden, Dresden, Germany; Public Health Agency of Barcelona, Spain

## Abstract

**Objective:**

Exposure to war can negatively affect health and may impact on healthcare costs. Estimating these costs and identifying their predictors is important for appropriate service planning. We aimed to measure use of health services in an adult population who had experienced war in the former-Yugoslavia on average 8 years previously, and to identify characteristics associated with the use and costs of healthcare.

**Method:**

War-affected community samples in Bosnia-Herzegovina, Croatia, Kosovo, FYR Macedonia, and Serbia were recruited through a random walk technique. Refugees in Germany, Italy and the UK were contacted through registers, organisations and networking. Current service use was measured for the previous three months and combined with unit costs for each country for the year 2006/7. A two-part approach was used, to identify predictors of service use with a multiple logistic regression model and predictors of cost with a generalised linear regression model.

**Results:**

3,313 participants were interviewed in Balkan countries and 854 refugees in Western European countries. In the Balkan countries, traumatic events and mental health status were related to greater service use while in Western countries these associations were not found. Participants in Balkan countries with post traumatic stress disorder (PTSD) had costs that were 63% higher (p = 0.005) than those without PTSD. Distress experienced during the most traumatic war event was associated with higher costs (p = 0.013). In Western European countries costs were 76% higher if non-PTSD anxiety disorders were present (0.027) and 63% higher for mood disorders (p = 0.006).

**Conclusions:**

War experiences and their effects on mental health are associated with increased health care costs even many years later, especially for those who stayed in the area of conflict. Focussing on the mental health impact of war is important for many reasons including those of an economic nature.

## Introduction

Exposure to war can negatively affect mental health. Studies show that exposure to prolonged war-related traumatic events is associated with high prevalence rates of mental disorders [1], [2], and greater than those found in the general population not affected by war [3], [4], [5]. Many studies focus on refugees in Western countries rather than the people who stay in the area of conflict [6], who are usually the vast majority of the affected population. Furthermore, these consequences of war could last several years after the ending of the conflict and these long term effects are likely to have economic consequences due to the need for services and care from family members [7], [8]. Assessment of these costs is important given limited health care funds, especially in lower income countries which are disproportionately affected by war.

The collapse of Yugoslavia in the early 1990s precipitated the worst armed conflict in Europe since 1945. War activities occurred at different places in former Yugoslavia between 1991 and 2001 [9]. Whilst most affected people stayed in the area of conflict, large numbers sought residence in Western Europe [10]. For those who experienced war and stayed in the Balkan countries, Priebe et al have recently reported high rates of posttraumatic stress disorder (prevalence rates ranged from 10.6% to 35.4%), mood disorders (12.1% to 47.6%,) and other anxiety disorders (from 15.6% to 41.8%) [11].

This paper has two aims. First, to assess the long-term economic consequences of war through the measurement of the current use and cost of health services among a population who experienced war-related traumatic events in the former Yugoslavia. Second, to identify individual-level factors that explain the variation in healthcare costs and assess whether these factors differ between individuals who stayed in the war regions and those who took refuge in other countries.

## Methods

### Setting and sample

The rationale and methods have been described in detail elsewhere and so only a summary is included here [11], [12], [13], [14]. Participants were recruited to a multi-centre epidemiological community-based study carried out in five in the Balkan region (Bosnia and Herzegovina, Croatia, Kosovo, FYR Macedonia, and Serbia) and in three West European countries which received refugees from the area (Germany, Italy and UK). Areas in each Balkan country were randomly selected if they had been directly exposed to war. Every fourth house in randomly selected streets was chosen and an eligible adult with a birthday closest to the interview date was interviewed. Potential participants from Italy and Germany were identified from resident registers and ‘snowballing’ (i.e. new participants were referred by existing participants). In the UK, participants were recruited through community organisations and snowballing. Inclusion criteria were: born in former Yugoslavia, age 18–65, experienced at least one war-related event with the last event at or after the age of 16, and no mental impairment due to brain injury or organic cause. The potentially traumatic war-related experience was established using a screening list containing 20 stressful events that people may have experienced during wartime (eg, shelling, sexual assault, or combat).

### Measures

Face-to-face interviews were conducted in 2005 and 2006 with data collected on demographic characteristics, potentially traumatic experiences before, during and after the war assessed on an adapted 24 item version of the Life Stressor Checklist-Revised (LSC-R) [15], and current mental disorders using the Mini International Neuropsychiatric Interview (MINI) [16]. Service use was measured using an adapted version of the Client Service Receipt Inventory [17], with participants asked to provide details of services used during the previous three months. Services included primary and secondary healthcare and medication. Written informed consent was obtained from all participants before the interview. The study was approved by the Royal Free Medical School Research Ethics Committee (REC reference number 04/QO501/118).

### Service Costs

The service use data were combined with unit costs for each country for the year 2006/2007. These country specific unit costs were derived (data available in online version) as follows: (i) UK unit costs were obtained from a range of national sources [18], [19] and (ii) transformed into international dollars for the year 2006 using purchasing power parity data [20]; (iii) the ratio between health costs in the UK and each other country was calculated using data from the World Health Organisation [21] and (iv) this ratio was applied to the unit costs to derive country specific figures. Data on medication were particularly detailed and extensive and we therefore calculated medication costs for a random sample of 5% (n_total_ = 208) of the individuals included in the study for each country. The mean pharmaceutical cost obtained for each country was used to attribute a pharmaceutical cost to the rest of the sample.

### Analyses

Mean service costs during the three months prior to the interview date were categorised as: community health care (general practitioner, primary care nurse, social worker, counsellor and other community professional), mental health specialist services (psychiatry, psychology and psychotherapy) and non-mental health specialist services. Total service costs were calculated by adding the cost of medication to the health care costs.

Regression models were used with the objective of identifying variables that explained the variation in resource use between participants. Cost data such as these are often characterised by a large number of zeros (due to non-use) and skewed distributions (due to a small number of high service users). A two-part approach was used to first identify predictors of service use and then to identify predictors for costs among users [22]. The first part was a multiple logistic regression model which took use (yes/no) of healthcare services as the dependent variable. The second part was a generalised linear model which took healthcare service costs for those using services as the dependent variable and used a log link and gamma distributed errors to deal with the skewed distribution [22]. Separate regression analyses for individuals who stayed in the war area (Balkans) and for those who migrated to Western countries were carried out, controlling for country of residence in the two parts of the model.

The independent variables included in the models were categorised as: *pre-war variables* (age, gender, education level and number of traumatic events experienced before the war), *war-related variables* (number of traumatic events during the war, time since the most traumatic war experiences, level of distress on a 5-point Likert scale, |[0 = not at all, to 4 = extremely], experienced at the time of the most traumatic war event and active participation in war) and *post-war variables* (employment, marital status and number of traumatic events after the war).

For estimating differences between countries in the logistic analysis, the overall mean of the five Balkan countries and three Western European countries respectively was taken as the reference categories using effect coding. For the second part of the model, binary country variables were entered with UK used as the reference category. Finally, the presence or absence of four types of mental disorders (PTSD, any other anxiety disorders, mood disorder, and substance abuse disorder) were included.

## Results

In the Balkan countries, 70.1% of eligible participants were interviewed. In Western countries, 52.9% of those people who responded to invitation letters were interviewed, whilst response rates for snowball sampling could not be established. A total of 3313 participants were interviewed in Balkan countries and 854 in West European countries. Socio-demographic characteristics, trauma-related variables and prevalence of different mental disorders are reported in Table 1. Working status of the individuals varied notably between countries, with the lowest level of employment in Germany and the highest in Italy. Participants with no formal or up to primary education were most common in Kosovo, FYR Macedonia and Bosnia and Herzegovina. Participants from Croatia and Bosnia and Herzegovina were most likely, and those in Kosovo least likely, to have been involved in combat. The number of war-related events experienced was greatest in Germany, the UK and Bosnia and Herzegovina with an average time since the most traumatic event of 8.6 years for the whole sample. There was also variation in the occurrence of mental health disorders with mood, anxiety and substance abuse being most common in Germany and least common in FYR Macedonia.

**Table 1:** Sample characteristics.

	Total	UK	Germany	Italy	Serbia	Croatia	FYR Macedonia	BiH	Kosovo
**Total participants**	4167	302	255	297	637	727	661	640	648
**Female gender (%)**	53.3	55.6	52.2	46.1	57.0	54.1	47.4	53.9	57.0
**Age** (mean)	42.3	43.9	41.9	38.9	39.9	45.8	40.1	46.2	39.4
**No or primary education (%)**	28.7	22.5	23.1	20.5	8.8	22.6	46.3	32.5	42.1
**Married/cohabiting (%)**	71.5	73.0	74.1	81.5	59.8	72.4	76.7	66.1	75.6
**Employed (%)**	37.1	29.1	22.7	71.4	48.8	38.5	29.2	37	26.1
**Combat involvement (%)**	18.5	14.9	23.1	29.6	9.1	32.2	7.0	33.6	3.9
**Number of war-related traumatic events (mean)**	4.7	7.4	7.8	5.2	2.4	4.6	1.9	6.9	5.1
**Time since war trauma (mean years)**	8.6	10.6	11.0	9.8	6.3	11.9	4.0	11.3	6.7
**Any mood disorder (%)**	31.7	45.1	57.4	30.0	35.9	25.9	12.1	22.7	47.6
**PTSD (%)**	22.8	28.8	54.9	18.9	18.8	18.0	10.6	35.3	18.2
**Other anxiety disorder (%)**	12.8	13.6	5.9	11.4	20.7	12.0	5.0	6.1	23.6
**Substance abuse disorder (%)**	4.8	1.7	11.8	0.7	9.0	6.5	0.6	5.5	2.8

Among Western European countries, participants from Germany reported higher use of specialised services, both psychiatric and non-psychiatric (Table 2). Participants from Bosnia and Herzegovina used specialised mental health services three- to ten-fold more than those in the other Balkan countries. FYR Macedonia and Kosovo had lowest use of all services.

**Table 2:** Use of healthcare services during previous 3 months (%) and mean (SD) number of contacts.

	Community health care		Psychiatric services		Other specialist health services		Medication	Any health care service
Country	%	Mean (Sd)	%	Mean^1^ (Sd)	%	Mean^1^ (Sd)	%	%
**UK**	75.2	3.33 (5.07)	10.3	0.22 (0.98)	52.3	1.32 (2.62)	67.2	88.7
**Germany**	62.0	3.63 (5.66)	45.1	3.01 (5.46)	71.0	5.04 (7.84)	83.9	93.7
**Italy**	50.2	2.23 (5.79)	4.0	0.09 (0.63)	45.8	1.33 (2.90)	70.0	81.5
**Serbia**	44.6	2.88 (9.91)	6.4	0.17 (0.92)	48.4	1.86 (3.68)	72.2	82.1
**Croatia**	62.9	3.75 (11.94)	7.4	0.22 (0.99)	43.9	1.09 (2.08)	71.7	82.4
**BiH**	58.4	2.91 (4.99)	20.6	0.72 (1.85)	47.5	1.61 (2.84)	72.5	80.9
**FYR Macedonia**	40.1	1.51 (3.60)	3.9	0.07 (0.45)	34.5	1.24 (4.45)	59.6	69.9
**Kosovo**	32.3	0.94 (2.16)	1.9	0.08 (0.73)	26.1	1.32 (2.62)	54.6	61.9

1; excluding hospital admissions.

These differences observed in the use of services when combined with the unit costs for each country resulted in substantial variations in costs (Table 3). Not surprisingly, costs were higher in the Western countries than the Balkan ones. Costs of all services were highest in Germany, with the exception of medication, which was most expensive in the UK. Total costs in Germany were over twice as high as those in Italy or the UK. Among Balkan countries, the highest costs were in Serbia and the lowest in FYR Macedonia and Kosovo. The distribution of the costs between service categories also varied. Medication costs accounted for substantially more of the total in the UK compared to all other countries.

**Table 3:** Service costs during previous 3 months (international dollars, 2006).

	Community health care		Psychiatric services		Other specialist health services		Medication		Total
	Mean (SD)	%^1^	Mean (SD)	%^1^	Mean (SD)	%^1^	Mean (SD)	%^1^	Mean (SD)
**UK**	172 (380)	33.3	44 (199)	8.4	217 (696)	41.9	85 (67)	16.4	518 (863)
**Germany**	249 (441)	11.7	648 (1174)	30.5	1,176 (3029)	55.3	53 (33)	2.5	2126 (3409)
**Italy**	140 (382)	25.4	18 (127)	3.3	380 (1792)	68.9	13 (10)	2.4	551 (1916)
**Serbia**	39 (293)	21.3	7 (39)	4.0	136 (770)	73.7	2 (1)	1.1	185 (867)
**Croatia**	57 (358)	23.3	71 (616)	29.1	105 (369)	43.0	11 (8)	4.7	243 (816)
**BiH**	35 (75)	18.1	51 (277)	26.5	95 (391)	49.3	12 (10)	6.1	194 (509)
**FYR Macedonia**	22 (88)	23.5	4 (27)	4.4	64 (357)	67.9	4 (4)	4.2	95 (384)
**Kosovo**	14 (44)	22.2	9 (183)	14.9	38 (183)	60.4	2 (2)	2.6	64 (265)

1; percentage of the total cost.

The logistic regression models show that there were key differences between Western and Balkans countries in the factors related to using healthcare services (Table 4). In the Western countries age was positively related to service use whilst involvement in combat predicted lower use. In the Balkan countries, the number of potentially traumatic events suffered before, during, and after the war increased the likelihood of using services. Men were less likely to receive care than women. As in the West, age was positively and combat experience negatively related to service use, and PTSD, mood and other anxiety disorders predicted higher use.

**Table 4:** Logistic regression; factors that influence the probability of having used any healthcare service.

Western	Exp(B)	95% C.I.for EXP(B)		Balkan	Exp(B)	95% C.I.for EXP(B)	
**Mental disorders MINI assessment**				**Mental disorders MINI assessment**			
PTSD	2.011	0.974	4.155	PTSD*	2.727	2.006	3.707
Any other anxiety disorder	1.231	0.571	2.653	Any other anxiety disorder*	1.700	1.268	2.281
Mood disorder	1.492	0.807	2.759	Mood disorder*	1.514	1.184	1.935
Substance abuse disorder	0.815	0.216	3.071	Substance abuse disorder	1.208	0.773	1.887
**War context**				**War context**			
Number of war traumatic events (log)	1.037	0.601	1.790	Number of war traumatic events (log)*	1.302	1.011	1.677
Combat involvement*	0.541	0.304	0.964	Combat involvement*	0.735	0.556	0.971
Time since trauma	0.961	0.886	1.042	Time since trauma	0.969	0.891	1.053
Level of distress	0.956	0.498	1.834	Level of distress	0.920	0.775	1.092
**Pre-war context**				**Pre-war context**			
Age (years)*	1.026	1.001	1.052	Age (years)*	1.020	1.012	1.029
Gender (female reference category)	0.626	0.354	1.107	Gender (female reference category)*	0.456	0.374	0.556
Education (none or elementary reference category)				Education (none or elementary reference category)			
Secondary	0.497	0.241	1.022	Secondary	0.989	0.790	1.238
Tertiary	0.67	0.315	1.428	Tertiary	1.110	0.841	1.463
Number of prewar traumatic events (log)	1.295	0.854	1.965	Number of prewar traumatic events (log)*	1.253	1.023	1.534
**Post-war context**				**Post-war context**			
Number of post-war traumatic events (log)	0.985	0.633	1.533	Number of post-war traumatic events (log)*	1.737	1.388	2.175
No living with partner	1.309	0.733	2.339	No living with partner	0.906	0.737	1.114
Employed	1.143	0.670	1.951	Employed	0.900	0.744	1.089
**Countries**				**Countries**			
UK	0.893	0.627	1.273	Serbia	1.249	0.949	1.644
Germany*	1.684	1.093	2.593	Croatia*	1.590	1.079	2.344
Italy*	0.665	0.467	0.947	BiH	1.255	0.877	1.796
				FYR Macedonia	0.979	0.653	1.467
				Kosovo*	0.410	0.329	0.511
Constant	6.924			Constant	1.563		

*Variables statistically significant at p<0.05.

The model that examined the variation in costs among individuals using healthcare services explained 39.4.% of the observed variation in Western countries and 19.1% in Balkans countries (Table 5). The exponentiated coefficients are displayed, indicating the proportional impact on cost of a unit change in the independent variable. Participants in Balkan countries with PTSD had costs that were 63% higher (p = 0.005) than those without PTSD. The level of distress experienced at the time of the most traumatic war event (p = 0.013) and mood (p = 0.004) disorders also predicted higher healthcare costs in this population. In Western European countries, presence of other anxiety disorders (p = 0.027) and mood disorders (p = 0.006) were related to costs that were 76% and 63% respectively higher compared to the absence of these disorders. Employed individuals had lower healthcare costs than those out of work (p = 0.024).

**Table 5:** Generalised linear models; factors that influence the variation of costs among individuals who used healthcare services.

Western	Exp (coeff)	95% Conf.Interval		Balkan	Exp (coeff)	95% Conf.Interval	
**Mental disorders MINI assessment**				**Mental disorders MINI assessment**			
PTSD	1.408	0.971	2.042	PTSD*	1.628	1.155	2.294
Other anxiety disorders*	1.767	1.067	2.927	Other anxiety disorders	0.963	0.671	1.384
Mood disorder*	1.630	1.154	2.303	Mood disorder*	1.732	1.301	2.306
Substance abuse disorder	0.960	0.471	1.956	Substance abuse disorder	0.763	0.447	1.302
**War context**				**War context**			
Number of war traumatic events	1.006	0.959	1.055	Number of war traumatic events	0.957	0.895	1.023
Combat involvement	1.081	0.704	1.660	Combat involvement	0.986	0.651	1.494
Time since trauma	1.011	0.961	1.063	Time since trauma	0.908	0.820	1.004
Level of distress	0.949	0.613	1.470	Level of distress*	1.341	1.063	1.692
**Pre-war context**				**Pre-war context**			
Age (years)	1.007	0.993	1.022	Age (years)	1.008	0.997	1.019
Gender (female reference category)	0.806	0.569	1.140	Gender (female reference category)	1.281	0.959	1.710
Education (years)	0.996	0.959	1.036	Education (years)	0.967	0.930	1.006
Number of prewar traumatic events	1.055	0.948	1.175	Number of prewar traumatic events	1.115	0.987	1.259
**Post-war context**				**Post-war context**			
Number of post-war traumatic events	1.095	0.967	1.239	Number of post-war traumatic events	1.147	0.981	1.340
No living with partner	0.859	0.605	1.220	No living with partner	0.982	0.744	1.296
Employed*	0.659	0.459	0.947	Employed	0.861	0.659	1.125
**Countries** (UK reference)				**Countries** (Serbia reference)			
Germany*	3.152	2.201	4.512	Croatia*	2.514	1.229	5.143
Italy	1.405	0.928	2.127	BiH*	2.201	1.048	4.624
				FYR Macedonia*	0.590	0.358	0.974
				Kosovo*	0.573	0.347	0.946
Constant	320.043	52.722	1942.801	Constant	76.292	21.479	270.983

*Variables statistically significant at p<0.05.

## Discussion

Wars have devastating effects for the communities that experience them, and these consequences can last long after the conflict is ended. The data presented here, relating to the countries of former-Yugoslavia, on the recent use and costs of healthcare services and the factors that influence them highlight these long-term implications of wars. Based on smaller and more selective samples, we have previously reported the costs of services for those who have not actively sought specialist treatment and those who have been in receipt of such care [23], [24]. This is the largest study we are aware of to assess the healthcare service use and costs for populations affected by war, regardless of whether they actively participated in the war or not and if they stayed in the conflict zone or migrated. War experiences and their effects on mental health are associated with increased health care costs even many years later, especially for those who stayed in the area of conflict.

Whilst all participants had some shared experiences, some cost differences at the country and individual level were to be expected given the particular service systems in each country and the differences in demographic and clinical characteristics between participants as well as the degree to which traumatic events were experienced. Such differences might have had an effect on the variation in the prevalence of mental disorders found between the countries, which in turn might impact on the use of healthcare services.

One of the key reasons for differences in resource use is likely to be the availability of services. Such availability is largely determined by a country's economic status. It is perhaps not surprising therefore that costs are highest in Germany and lowest in FYR Macedonia and Kosovo. In addition, other individual characteristic such as gender and health status of the individuals might play a role in explaining the large variation found between countries in the use of healthcare services. Additionally, the results for participants in Germany could be partially explained by the fact that the resident permit status for refugees and asylum seekers is partly linked to mental health status. Indeed, higher costs for refugees settled in Germany were also found in a previous study [24].

There were key differences between Western and Balkans countries in the factors that influence use and costs of healthcare services in this population. In respect to predictors of use in the Western countries age and involvement in combat were related to use. The latter variable could be linked to the gender of the individuals given that very few women were involved in combat. Indeed, in analyses excluding the combat involvement variable, the gender of the individuals became significant (available from authors). In a previous study from the area, age was found to have a positive impact on resource use [23]. Neither war-related traumatic experiences nor mental health status were related to use of healthcare services. For the population who stayed in the Balkan countries, the results are very different. Age and gender did affect access to these services but in addition lifetime traumatic experiences and mental health status were also significant predictors. PTSD in particular was associated with greater use of services. In previous work looking at those not seeking treatment, PTSD was found not be associated with costs [23]. The sample here includes both treatment seekers and those not receiving care, which may explain the stronger link.

These findings might be explained by more developed and well funded health systems in Western countries. This explanation of different accessibility might be supported by the fact that healthcare private out-of pocket expenditure accounts for a disproportionate high share of total healthcare expending in some of the Balkan countries [25].

Once individuals have accessed healthcare in Balkan countries, the cost of services is related to clinical health needs and the severity of the traumatic experiences. Participants with PTSD or mood disorders had significantly higher costs than those without these conditions. These findings reinforce the positive relationship between war events and higher costs given the established relationship between the prevalence of these disorders and these types of potentially traumatic events [11]. This relationship is also found for individuals who migrated, although there PTSD is not statistically significant and that no war-related variable predicts costs (as is the case of the level of distress variable in Balkans countries).

### Strengths and limitations

This study measured service use and costs for populations affected by war using a consistent methodology across several countries. A random walk technique applied in Balkan countries ensured that the findings are representative for large populations in the war-affected areas. The study included civilians and people with combat experience, and people who stayed in the area of conflict and refugees. Further strengths are that all interviewers were well-trained researchers with a relevant professional background, were familiar with the given local context, and spoke the mother tongue of the interviewees.

There were a number of limitations to the study. First, the sampling procedure applied in Western European countries was less rigorous, possibly leading to less representative samples. The samples in these countries were also smaller, resulting in a possible larger random error of findings. Second, most variation in resource use was left unexplained. This could be due to some factors being missing from the models, to measurement error or to random influences. Third, service use was measured for a retrospective three-month period and relied on participant recall. This was necessary given that access to administrative records was not available (and few records would contain the breadth of service use data required). However, it is a recognised method and a number of studies [26], [27] have been conducted to support its reliability. Fourth, medication costs were calculated for 5% of the sample and then mean costs applied to the remaining participants. This was necessary given the extent of the medication data but it does introduce a level of inaccuracy. However, medication is likely to account for only a small amount of the overall costs. Fifth, service use was measured over a period that was some time after the wars in the region. This has its advantages in that the longer-term consequences of the wars can be measured but a limitation is that it does not tell us what has happened in the intervening period. Sixth, given that people with severe brain injuries were excluded from the analysis and information could not be collected for institutionalised individuals given the sampling procedure, it might be the case that health care costs were underestimated. Finally, the study did not include a control group. The entire sample experienced at least one war-related event. Therefore no information was available on the use and cost of healthcare resources by those who were not exposed to war. However, the use of the multivariate models allows us to identify the impact of the quantity of war-related events on costs.

### Conclusions

This is the largest study to date that has examined healthcare costs related to war. Past experiences in war are associated with current healthcare costs, especially for those who stayed in the area of conflict. Health care costs are substantially increased independently of country specific variations and mental health plays a major role in this. These disorders not only represent substantial distress for those suffering from them, but may also place a significant burden on healthcare systems. Post conflict policies and support for war affected countries should consider that health care costs may remain raised for many years.
